# Functional and morphological determinants of left ventricular fibrosis in patients with mitral annular disjunction in cardiac magnetic resonance imaging

**DOI:** 10.1093/ehjimp/qyaf120

**Published:** 2025-09-30

**Authors:** Martin F Reiner, Daniel Escribano-García, Federica Guidetti, Verena C Wilzeck, Rabea Schlenker, Andrea Biondo, Firat Duru, Frank Ruschitzka, Gianluigi Savarese, Hatem Alkadhi, Robert Manka

**Affiliations:** Diagnostic and Interventional Radiology, University Hospital Zurich, University of Zurich, Rämistrasse 100, Zurich 8091, Switzerland; Department of Cardiology, University Heart Center, University Hospital Zurich, Rämistrasse 100, Zurich 8091, Switzerland; Cardiology Department, Hospital Universitario Puerta de Hierro-Majadahonda, Madrid, Spain; Division of Cardiology, Department of Medicine, Karolinska Institutet, Stockholm, Sweden; Diagnostic and Interventional Radiology, University Hospital Zurich, University of Zurich, Rämistrasse 100, Zurich 8091, Switzerland; Department of Cardiology, University Heart Center, University Hospital Zurich, Rämistrasse 100, Zurich 8091, Switzerland; Institute for Biomedical Engineering, University and ETH Zurich, Rämistrasse 101, 8092 Zurich, Switzerland; Diagnostic and Interventional Radiology, University Hospital Zurich, University of Zurich, Rämistrasse 100, Zurich 8091, Switzerland; Department of Cardiology, University Heart Center, University Hospital Zurich, Rämistrasse 100, Zurich 8091, Switzerland; Diagnostic and Interventional Radiology, University Hospital Zurich, University of Zurich, Rämistrasse 100, Zurich 8091, Switzerland; Department of Cardiology, University Heart Center, University Hospital Zurich, Rämistrasse 100, Zurich 8091, Switzerland; Center for Integrative Human Physiology, University of Zurich, Zurich, Switzerland; Department of Cardiology, University Heart Center, University Hospital Zurich, Rämistrasse 100, Zurich 8091, Switzerland; Department of Cardiology, Center for Translational and Experimental Cardiology (CTEC), Zurich University Hospital, University of Zurich, Schlieren, Switzerland; Division of Cardiology, Department of Medicine, Karolinska Institutet, Stockholm, Sweden; Diagnostic and Interventional Radiology, University Hospital Zurich, University of Zurich, Rämistrasse 100, Zurich 8091, Switzerland; Diagnostic and Interventional Radiology, University Hospital Zurich, University of Zurich, Rämistrasse 100, Zurich 8091, Switzerland; Department of Cardiology, University Heart Center, University Hospital Zurich, Rämistrasse 100, Zurich 8091, Switzerland; Institute for Biomedical Engineering, University and ETH Zurich, Rämistrasse 101, 8092 Zurich, Switzerland

**Keywords:** fibrosis, mitral annular disjunction, mitral valve prolapse, CMR

## Abstract

**Aims:**

The study investigates determinants of left ventricular (LV) fibrosis in patients with inferolateral mitral annular disjunction (MAD).

**Methods and results:**

This single centre retrospective cohort study included 111 patients [median age 52 years (25th–75th percentile 36–69), 47% female] with inferolateral MAD that underwent cardiac magnetic resonance imaging. Mid-myocardial replacement fibrosis of the basal inferolateral LV wall was present in 27 (24%) patients. Patients with fibrosis were older [63.0 years (25th–75th percentile 53.0–70.0) vs. 47.5 years (25th–75th percentile 34.0–67.5), *P* = 0.011], more likely to have mitral valve prolapse (MVP) [24/27 (89%) vs. 58/84 (69%), *P* = 0.041], had larger isolated posterior prolapse size [5.0 mm (25th–75th percentile 5.0–5.0) vs. 3.0 mm (25th–75th percentile 2.0–4.0), *P* = 0.041], were more likely to have bileaflet MVP [17/27 (63%) vs. 30/84 (36%), *P* = 0.013], had larger MAD distance [6.0 mm (25th–75th percentile 5.0–9.0) vs. 5.0 mm (25th–75th percentile 3.0–8.0), *P* = 0.018] and were more likely to exhibit an accelerated longitudinal motion of the basal inferolateral LV segment [20/27 (74%) vs. 26/84 (31%), *P* < 0.001]. Multivariable logistic regression revealed that age [odds ratio (OR) 1.06, confidence interval (CI) 1.02–1.10, *P* = 0.001] and accelerated longitudinal motion of the basal inferolateral LV segment (OR 11.10, CI 2.83–43.60, *P* = 0.001) were significantly associated with replacement fibrosis. Bileaflet MVP (OR 4.28, CI 1.51–12.13, *P* = 0.006) and MAD size (OR 1.24, CI 1.01–1.52, *P* = 0.041) were associated with an accelerated motion of the basal inferolateral LV segment.

**Conclusion:**

Age and accelerated longitudinal motion of the basal inferolateral LV segment are independent determinants of replacement fibrosis of the basal inferolateral wall in patients with MAD. The presence of bileaflet MVP and MAD size are significantly associated with this accelerated movement pattern.

## Graphical Abstract

**Graphical Abstract:**
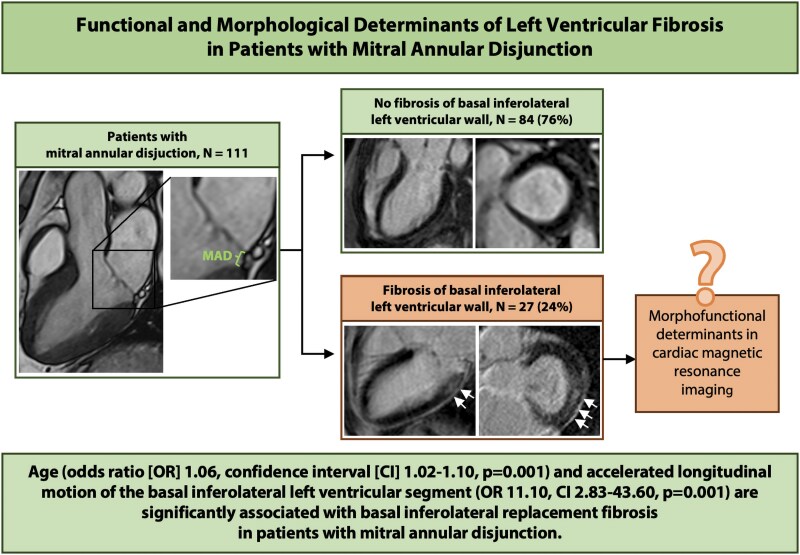

## Introduction

Mitral annular disjunction (MAD) is characterized by a separation of the mitral valve hinge point and the left ventricular (LV) myocardium.^1,2^ It is associated with mitral valve prolapse (MVP) in 78%^3^ and conversely, 30% of MVP patients present with MAD.^4^ The prognosis of isolated MVP is comparable to the general population but mortality increases substantially in the presence of moderate mitral insufficiency or higher and reduced LV function.^5^ In addition, a subset of patients with MVP is at increased risk for ventricular arrhythmias^6^ and sudden cardiac death,^7^ a condition classified as arrhythmic MVP.^8^ The risk for arrhythmias in MVP is substantially increased in the presence of MAD^3,9^; likewise, patients with MAD without MVP are at substantial risk for ventricular tachycardia and cardiac arrest emphasizing the arrhythmic potential of MAD.^3^ It has been hypothesized that the underlying mechanisms of arrhythmias encompass the combination of LV fibrosis serving as the arrhythmic substrate and mechanical stretch acting as a triggering factor.^10^ Indeed, MAD contributes to an abnormal movement of the inferobasal LV wall as well as the mitral anulus, referred to as curling, which is believed to increase mitral valve traction and myocardial stretch.^10,11^ In addition, LV fibrosis is more likely to occur in MVP patients with MAD.^11^ Indeed, fibrotic areas of the mitral annular region were identified by electroanatomical mapping as origin of sustained ventricular arrhythmias^12^ and fibrosis of the inferobasal LV wall predicts ventricular arrhythmias.^13^ However, determinants of fibrosis of the basal inferolateral LV myocardium in patients with MAD remain unknown.

In this study of 111 patients with MAD undergoing cardiac magnetic resonance (CMR) imaging, we investigated morphological and functional determinants of fibrosis of the basal inferolateral LV segment, a well-described source of ventricular arrhythmias.

## Methods

### Study population

**Figure 1:**
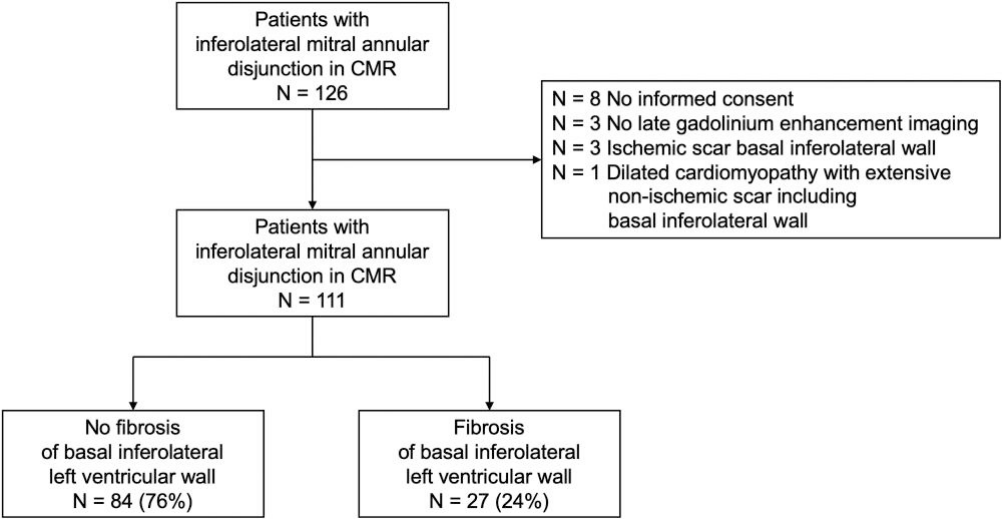
Flow chart patient selection. CMR, Cardiac magnetic resonance.

Reports of in- and out-patients who received CMR imaging between January 2020 and February 2024 were screened for the presence of inferolateral MAD. Fifteen patients were excluded [8: no informed consent, 3: no late gadolinium enhancement (LGE) imaging, 3: ischaemic scar of the LV inferolateral basal wall, 1: dilated cardiomyopathy with extensive scaring including the basal inferolateral LV wall] and 111 patients were finally included in the current study (*Figure 1*). None of the included patients had infiltrative cardiomyopathy such as sarcoidosis or amyloidosis. The study was approved by the ethic committee (Kantonale Ethikkommission Zürich, Nr. 2015-0582) and conducted in accordance with the declaration of Helsinki. All study participants provided written informed consent. This study adheres to STROBE guidelines and fulfils the minimum requirements of the CODE-EHR framework.

### Baseline characteristics and CMR imaging

**Figure 2:**
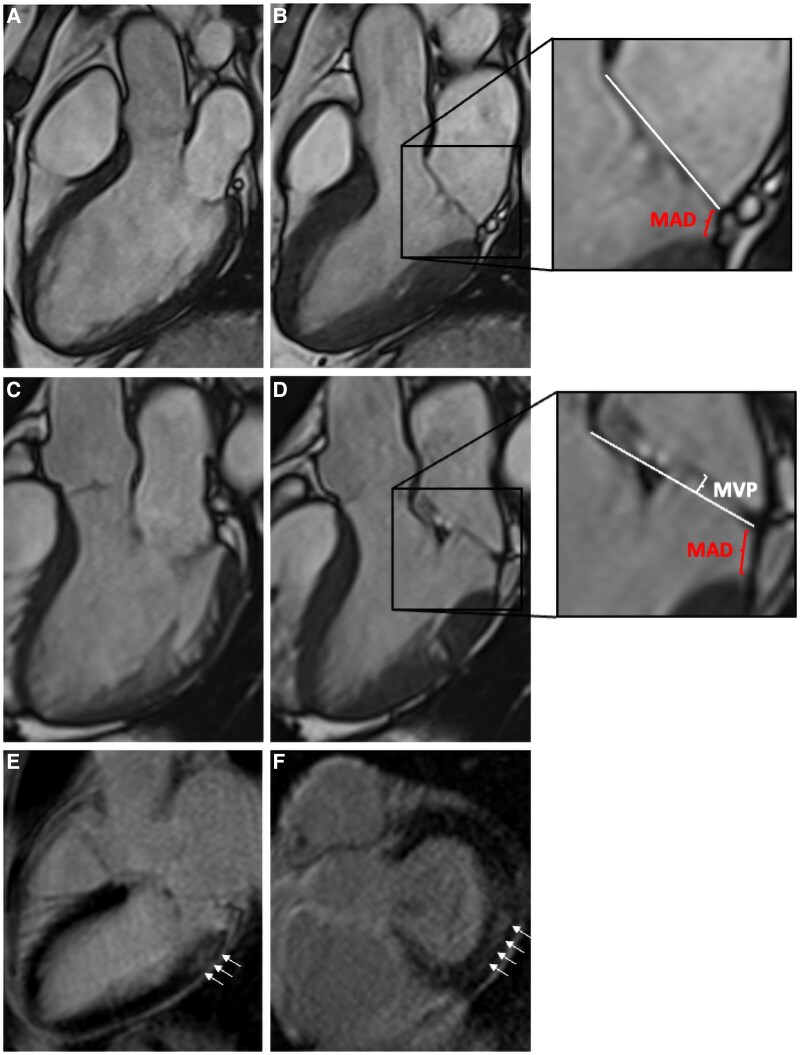
CMR protocol. CMR imaging of a patient with MAD in 3-chamber view in diastole (*A*) and systole (*B*), in a patient with both MAD and posterior MVP in 3-chamber view in diastole (*C*) and systole (*D*). LGE imaging showing replacement fibrosis in the basal inferolateral LV myocardium in 3-chamber view (white arrows) (*E*) and corresponding short axis view (white arrows) (*F*). LV, left ventricular; MAD, mitral annular disjunction; MVP, mitral valve prolapse.

**Figure 3:**
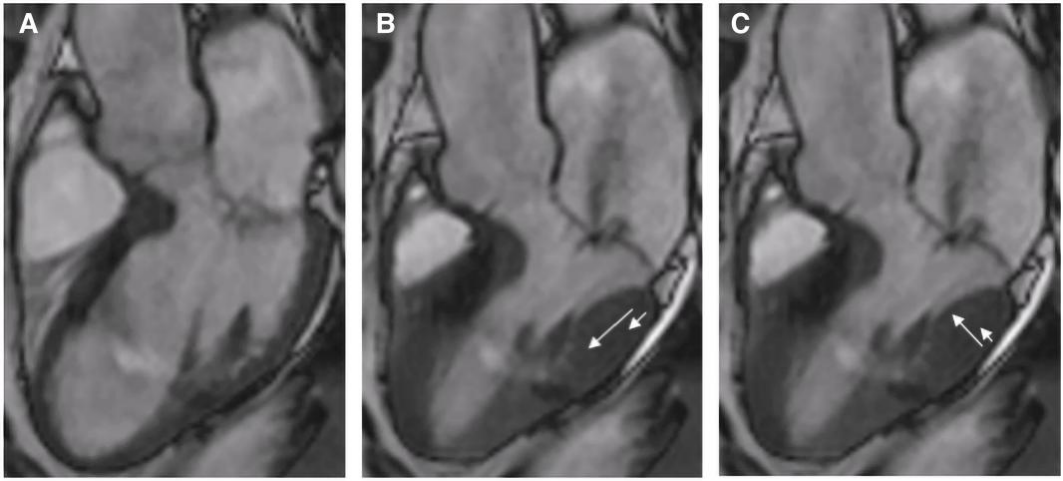
CMR imaging depicting accelerated longitudinal and radial motion of the basal inferolateral LV segment during systole. CMR imaging of a patient with MAD in 3-chamber view during diastole (*A*) as well as accelerated longitudinal (*B*) and radial (*C*) motion of the basal inferolateral LV segment during systole (depicted by white arrows). MAD, mitral annular disjunction.

Reports of CMR studies were reviewed for the presence of MAD. CMR images were then analysed for fibrosis, MAD distance, presence of anterior and posterior prolapse including prolapse size as well as longitudinal and radial displacement of the basal inferolateral LV wall. The remaining parameters were obtained from the report. Mid-myocardial replacement fibrosis was identified by visual assessment of mid-myocardial LGE of the basal inferolateral LV wall present in both 3-chamber view and basal short axis view (*Figure 2*). MAD distance [mm] was measured in systole in 3-chamber view as distance from the mitral valve hinge point to the LV myocardium^14^ (*Figure 2*). Patients with juxtaposition of the billowing posterior leaflet against the atrial wall, which mimics the appearance of MAD during systole, were not included.^15^ MVP was defined as systolic atrial displacement of the anterior and/or posterior mitral leaflet ≥ 2mm^16^ and measured in 3-chamber view^17^ (*Figure 2*). Given that echocardiographic studies using tissue Doppler imaging demonstrated a higher prevalence of malignant arrhythmias in MVP patients with accelerated longitudinal systolic movement of the basal anterolateral wall^18^ and that ventricular arrhythmias are more frequent in MVP/MAD patients with replacement fibrosis, which predominantly affects the basal inferolateral wall,^11,13^ we studied the movement pattern of this specific wall segment and its association with LV fibrosis. Longitudinal and radial displacement of the basal inferolateral LV wall was judged as homogeneous (*Figure 3* and Supplementary data online, *Video S1*) or accelerated (*Figure 3* and Supplementary data online, *Video S2*) by visual impression by two independent investigators blinded for the presence or absence of fibrosis.

### Statistical analysis

Normal distribution was tested using Shapiro–Wilk test. Categorical data are expressed as number and percentage of population. Normal distributed variables are expressed as mean [± standard deviation (SD)] and non-normal distributed variables as median (25th–75th percentile) Differences between groups with calculated using Pearson's chi-squared test for categorical variables and either a one-way ANOVA *F*-test (for normally distributed continuous variables) or Wilcoxon rank-sum test (for non-normally distributed continuous variables), as appropriate for continuous variables. Inter-rater reliability for accelerated longitudinal motion and accelerated radial motion of the basal inferolateral LV wall was analysed by Cohen’s Kappa statistics. Univariable and multivariable logistic regression models were used in the overall study population to identify patient characteristics independently associated with fibrosis of the basal inferolateral LV segment. In addition, univariable and multivariable logistic regression models were used to identify morphological parameters independently associated with accelerated longitudinal motion of the basal inferolateral LV wall. Variables were included in the multivariable logistic regression if we observed an association in the previously performed univariable logistic regression. Results are reported as odds ratios (ORs) and 95% confidence intervals (CI). Statistical analyses were performed by Stata 15.0 (Stata Corp LLC, College Station, TX, USA). A *P*-value ≤ 0.05 was considered statistically significant.

## Results

### Study population

The study included 111 participants with inferolateral MAD. Median age was 52 years (25th–75th percentile 36–69) and 52 (47%) participants were female. Median inferolateral MAD size was 6.0 mm (25th–75th percentile 4.0–8.0). Twenty-nine (26%) participants had isolated MAD without MVP, whereas 82 (74%) had MAD with concomitant MVP. Isolated anterior MVP was present in 3 (3%), isolated posterior MVP in 32 (29%) and bileaflet MVP in 47 (42%) participants. Mid-myocardial replacement fibrosis of the basal inferolateral LV wall was detected in 27 (24%) patients. We detected an accelerated longitudinal motion of the basal inferolateral LV wall in 46 (41%) and an accelerated radial motion of the basal inferolateral LV wall in 27 (24%) participants (*Table 1*). The indications for CMR in the study population were ischaemia (23 patients), ischaemia or structural heart disease (29 patients), structural heart disease (31 patients), inflammatory heart disease (1 patient), structural or inflammatory heart disease (13 patients), graduation of mitral insufficiency (4 patients), sarcoidosis (4 patients), adult congenital heart disease (5 patients), and haemochromatosis (1 patient).

**Table 1:** Baseline characteristics of patients with inferolateral MAD

Baseline characteristics	Total *N* = 111	No fibrosis *N* = 84	Fibrosis *N* = 27	*P*-value
Age [years], median (25th–75th percentile)	52.0 (36.0–69.0)	47.5 (34.0–67.5)	63.0 (53.0–70.0)	0.011
Female [%]	52 (47%)	42 (50%)	10 (37%)	0.24
Height [m], mean ± SD	1.73 ± 0.09	1.74 ± 0.10	1.71 ± 0.08	0.082
Weight [kg], median (25th–75th percentile)	66.0 (58.0–76.0)	64.0 (57.0–78.0)	68.0 (60.0–74.0)	0.45
Body mass index [kg/m^2^], median (25th–75th percentile)	22.5 (19.7–25.2)	22.0 (19.3–24.7)	22.9 (21.3–26.1)	0.063
Left atrial size [cm^2^], median (25th–75th percentile)	22 (19–26)	22 (19–25)	25 (20–30)	0.13
LVED volume [mL/m^2^], median (25th–75th percentile)	86.0 (77.0–107.0)	86.0 (77.5–100.5)	91.0 (73.0–118.0)	0.48
LV ejection fraction [%], mean ± SD	55.6 ± 6.5	56.1 ± 6.6	54.3 ± 6.1	0.20
LV mass [g/m^2^], median (25th–75th percentile)	44.0 (36.0–50.0)	43.0 (36.0–50.0)	46.0 (36.0–55.0)	0.38
MVP [%]	82 (74%)	58 (69%)	24 (89%)	0.041
Isolated anterior MVP [%]	3 (3%)	1 (1%)	2 (7%)	0.083
Isolated anterior MVP distance [mm], median (25th–75th percentile)	4.0 (2.0, 4.0)	4.0 (4.0, 4.0)	3.0 (2.0, 4.0)	0.48
Isolated posterior MVP [%]	32 (29%)	27 (32%)	5 (19%)	0.17
Isolated posterior MVP distance [mm], median (25th–75th percentile)	3.5 (2.0, 4.5)	3.0 (2.0, 4.0)	5.0 (5.0, 5.0)	0.041
Bileaflet MVP [%]	47 (42%)	30 (36%)	17 (63%)	0.013
MAD distance [mm], median (25th–75th percentile)	6.0 (4.0–8.0)	5.0 (3.0–8.0)	6.0 (5.0–9.0)	0.018
Accelerated longitudinal motion of the basal inferolateral LV segment [%]	46 (41%)	26 (31%)	20 (74%)	<0.001
Accelerated radial motion of the basal inferolateral LV segment [%]	27 (24%)	19 (23%)	8 (30%)	0.46

LV, left ventricular; LVED, left ventricular end-diastolic; MAD, mitral annular disjunction; MVP, mitral valve prolapse; SD, standard deviation.

### Determinants of replacement fibrosis of the basal inferolateral LV segment

Patients with basal inferolateral fibrosis were older [63.0 years (25th–75th percentile 53.0–70.0) vs. 47.5 years (25th–75th percentile 34.0–67.5), *P* = 0.011] and more likely to have MVP [24/27 (89%) patients vs. 58/84 (69%) patients, *P* = 0.041]. Patients with fibrosis had larger isolated posterior prolapse size [5.0 mm (25th–75th percentile 5.0–5.0) vs. 3.0 mm (25th–75th percentile 2.0–4.0), *P* = 0.041] and were more likely to have bileaflet MVP [17/27 (63%) vs. 30/84 (36%), *P* = 0.013]. In addition, median MAD distance was greater in patients with fibrosis, compared with patients without fibrosis [6.0 mm (25th–75th percentile 5.0–9.0) vs. 5.0 mm (25th–75th percentile 3.0–8.0), *P* = 0.018]. Finally, accelerated longitudinal motion of the basal inferolateral LV segment was significantly more frequently observed in patients with LV fibrosis [20/27 (74%) vs. 26/84 (31%), *P* < 0.001], while the prevalence of accelerated radial motion of the basal inferolateral LV segment was comparable between the groups (*Table 1*).

Univariable logistic regression revealed that age, presence of bileaflet MVP and accelerated longitudinal motion of the basal inferolateral LV segment were significantly associated with LV fibrosis (*Table 2*). Multivariable logistic regression including these variables, revealed that only age (OR 1.06, CI 1.02–1.10, *P* = 0.001) and accelerated longitudinal motion of the basal inferolateral LV segment (OR 11.10, CI 2.83–43.60, *P* = 0.001) significantly correlated with basal inferolateral fibrosis (*Table 2*).

**Table 2:** Odds ratio for replacement fibrosis of the basal inferolateral LV wall

Determinants of basal inferolateral LV fibrosis		
Univariable logistic regression	Odds ratio (95% CI)	*P*-value
Age^a^	1.04 (1.01–1.06)	0.008
LVED volume index	1.01 (0.99–1.03)	0.355
LV ejection fraction	0.96 (0.89–1.02)	0.201
LV mass index	1.03 (1.00–1.07)	0.082
MVP	3.59 (0.99–12.98)	0.052
Isolated anterior MVP	NA	NA
Isolated anterior MVP distance	NA	NA
Isolated posterior MVP	0.48 (0.16–1.40)	0.180
Isolated posterior MVP distance	0.96 (0.75–1.24)	0.758
Bileaflet MVP^a^	3.06 (1.24–7.52)	0.015
MAD distance	1.11 (0.97–1.26)	0.128
Accelerated longitudinal displacement^a^	6.37 (2.40–16.93)	<0.001
Accelerated radial displacement	1.44 (0.55–3.81)	0.462

Multivariable logistic regression	Odds ratio (95% CI)	*P*-value
Age	1.06 (1.02–1.10)	0.001
Bileaflet MVP	1.27 (0.38–4.22)	0.695
Accelerated longitudinal displacement	11.10 (2.83–43.60)	0.001

CI, confidence interval; LV, left ventricular; LVED, left ventricular end-diastolic; MAD, mitral annular disjunction; MVP, mitral valve prolapse; NA, not applicable due to low number of events.

^a^Variables included in multivariable logistic regression.

### Determinants of longitudinal motion of the basal inferolateral LV segment

After identifying accelerated longitudinal motion of the basal inferolateral LV segment as an independent determinant of LV fibrosis in this region, we further investigated determinants of this movement pattern. Patients presenting with accelerated longitudinal motion of the basal inferolateral LV segment had a larger median indexed LV end-diastolic volume [96.5 mL/m^2^ (25th–75th percentile 85.0–114.0) vs. 83.0 mL/m^2^ (25th–75th percentile 72.0–97.0), *P* < 0.001], were more likely to have MVP [43/46 (93%) vs. 39/65 (60%), *P* < 0.001] and bileaflet MVP [33/46 (72%) vs. 14/65 (22%), *P* < 0.001], had larger inferolateral MAD [7.5 mm (25th–75th percentile 6.0–9.0) vs. 4.0 mm (25th–75th percentile 3.0–6.0), *P* < 0.001] and were more likely to have fibrosis of the basal inferolateral LV segment [20/46 (43%) vs. 7/65 (11%), *P* < 0.001].

Univariable logistic regression showed that indexed LV end-diastolic volume, indexed LV mass, presence of bileaflet MVP and MAD size were associated with an accelerated longitudinal motion of the basal inferolateral LV segment (*Table 3*). Multivariable logistic regression including these variables revealed that only the presence of bileaflet MVP (OR 4.28, CI 1.51–12.13, *P* = 0.006) and MAD size (OR 1.24, CI 1.01–1.52, *P* = 0.041) significantly correlated with accelerated motion of the basal inferolateral LV segment (*Table 3*).

**Table 3:** Odds ratio for accelerated longitudinal motion of the basal inferolateral LV segment

Determinants of accelerated longitudinal displacement		
Univariable logistic regression	Odds ratio (95% CI)	*P*-value
Age	0.99 (0.97–1.01)	0.171
LVED volume index^a^	1.04 (1.02–1.06)	<0.001
LV ejection fraction	0.99 (0.93–1.05)	0.746
LV mass index^a^	1.04 (1.00–1.07)	0.034
MVP	9.56 (2.68–34.07)	0.001
Isolated anterior MVP	NA	NA
Isolated anterior MVP distance	NA	NA
Isolated posterior MVP	0.54 (0.23–1.29)	0.168
Isolated Posterior MVP distance	0.85 (0.68–1.07)	0.171
Bileaflet MVP^a^	9.25 (3.86–22.13)	<0.001
MAD distance^a^	1.46 (1.24–1.73)	<0.001

Multivariable logistic regression	Odds ratio (95% CI)	*P*-Value
LVED volume index	1.02 (0.98–1.06)	0.281
LV mass index	1.00 (0.95–1.05)	0.983
Bileaflet MVP	4.28 (1.51–12.13)	0.006
MAD distance	1.24 (1.01–1.52)	0.041

CI, confidence interval; LV, left ventricular; LVED, left ventricular end-diastolic; MAD, mitral annular disjunction; MVP, mitral valve prolapse; NA, not applicable due to low number of events.

^a^Variables included in multivariable logistic regression.

### Inter-rater agreement

Cohen’s kappa statistics was 0.85 for inter-rater agreement on accelerated longitudinal displacement of the basal inferolateral LV wall between observer 1 (M.F.R.) and observer 2 (D.E.-G.) and 0.95 for inter-rater agreement on accelerated radial displacement of the basal inferolateral LV wall between observer 1 (M.F.R.) and observer 2 (D.E.-G.) demonstrating excellent level of agreement for both observations.

## Discussion

In the current study of 111 patients with inferolateral MAD we investigated the association of morphological and functional CMR imaging parameters with fibrosis of the basal inferolateral LV segment, a well-described origin of ventricular arrhythmias.^12^ Patients with fibrosis of the basal inferolateral LV wall were significantly older, more likely to have MVP, had larger isolated posterior MVP, were more likely to exhibit bileaflet MVP, had greater inferolateral MAD size and were more likely to present with accelerated longitudinal motion of the basal inferolateral LV segment. In multivariable logistic analysis, only age and accelerated longitudinal motion of the basal inferolateral LV wall correlated significantly with the presence of basal inferolateral myocardial fibrosis. Our results for the first time demonstrate a significant association between the inferolateral LV movement pattern with local replacement fibrosis in patients with MAD. In addition, the association with age suggests that fibrosis may develop progressively over time in patients exhibiting this motion pattern. Finally, we found that the presence of bileaflet MVP and inferolateral MAD size were associated with accelerated longitudinal motion of the basal inferolateral LV segment suggesting that both, a pathological mitral valve apparatus and the extend of inferolateral MAD affect the motion of the basal inferolateral LV wall.

In this cohort of MAD patients, 26% had isolated MAD and 74% had both MAD and MVP, and fibrosis was detected in 24% of patients. These findings are in line with a prior investigation of unselected MAD patients showing isolated MAD in 22% and LV myocardial fibrosis in 17% of cases.^3^ Median inferolateral MAD distance was 6 mm, which was larger compared with previous studies reporting 3 mm^3,14^ suggesting that the studied population exhibits a more pronounced MAD. Former research reported an association between MAD/MVP and curling and it was hypothesized that curling may induce traction of the mitral leaflet subsequently leading to myocardial stretch and fibrosis.^10^ Initially described by Gilbert *et al*.,^19^ curling appears as systolic downward movement with limited or absent anterior motion of the mitral anulus resulting in a curled appearance.^19^ More recently, Basso *et al*. quantified curling by delineating a perpendicular line between the mitral anulus and the connection between the inferobasal LV wall and the left atrial wall/posterior mitral leaflet.^11^ However, despite the widespread use of this term in imaging, it lacks standardized definition^11,14,19^ and even more importantly, studies on curling and LV fibrosis are descriptive.^11^ Since replacement fibrosis predominantly affects the basal inferolateral myocardium in patients with inferolateral MAD, we aimed to study the movement pattern of this specific wall segment. While the majority of our cohort showed a homogenous longitudinal and radial movement of the basal inferolateral wall (see Supplementary data online, *Video S1*) others demonstrated an accelerated longitudinal or radial motion or both (see Supplementary data online, *Video S2*). This observation is supported by echocardiographic studies using tissue Doppler imaging and demonstrating accelerated longitudinal systolic movement of the basal anterolateral wall in certain patients with MVP, defined as Pickelhaube sign by Muthukumar *et al*.^18^ Interestingly, myocardial fibrosis and malignant arrhythmias were described more often in patients with MVP and Pickelhaube sign.^18^ Although this study examined the longitudinal movement pattern of the anterolateral LV wall by echocardiography and its potential association with arrhythmic events, it supports our findings, i.e. the correlation of an accelerated longitudinal motion of the basal inferolateral LV segment with fibrosis of this segment in MAD patients. Our findings provide a potential link between LV movement pattern, LV replacement fibrosis and the development of ventricular arrhythmias.

Multivariable logistic regression revealed that age and accelerated longitudinal motion of the basal inferolateral LV segment, but not the presence or extent of MVP, are independently associated with LV fibrosis suggesting a relevant link between mechanical force of the myocardial wall and replacement fibrosis. The significant association of age and fibrosis in MAD patients further suggests that a certain duration of exposure to increased myocardial force may be necessary for the development of fibrotic changes in the myocardium. This is in line with the observation that not all patients with accelerated longitudinal displacement showed LV fibrosis and is supported by the study of Essayagh et al. showing that the number of MAD patients affected by arrhythmic events increase with time.^20^ Whether accelerated motion and thus increased myocardial force of the basal inferolateral LV segment indeed elevates regional shear stress and consequently triggers fibrosis over time remains to be validated in experimental and prospective observational studies. In addition to replacement fibrosis, other mechanisms may be involved in the pathogenesis of arrhythmogenic MVP. Bui *et al*.^21^ reported higher prevalence of septal interstitial fibrosis, determined by post-contrast T1 times, in MVP patients with complex ventricular arrhythmias compared with patients without arrythmias.^21^ Whether interstitial fibrosis shows a homogeneous or inhomogeneous distribution in the LV of MAD patients and whether interstitial fibrosis is a precursor of replacement fibrosis at predisposed sites, such as the inferolateral basal LV wall, has to be investigated in future studies. In addition to fibrosis, inflammation may play a role in the pathogenesis of ventricular arrythmia. A small study of 20 patients with degenerative MVP and ventricular ectopy found subclinical inflammation in most of the patients and the side of inflammation correlated with replacement fibrosis.^22^ Finally, isolated myocardial hypertrophy of the basal inferolateral myocardium has been linked to MVP and is considered a consequence of increased wall stress; yet, its association with local fibrosis is inconsistent.^11,23^

We observed that the presence of bileaflet MVP and MAD size were independently associated with accelerated longitudinal LV motion, highlighting the impact of both—the extend of MAD and the presence of bileaflet MVP—on LV movement pattern. Depending on the localization and imaging modality, MAD is a frequent observation; a large CMR imaging study including over 2600 patients reported MAD at any location in 76%^14^ and a recent cardiac computed tomography study found MAD at any location in 96% of patients considering it a normal finding^24^; yet, inferolateral MAD was reported in only 5.1% by CMR imaging^14^ and in 11% by cardiac computed tomography^24^ making it an uncommon observation. The relevance of inferolateral MAD is further highlighted by its association with curling^14^ and its association with arrhythmic event.^3,12,20^ Dejgaard *et al*.^3^ reported an event rate of severe ventricular arrhythmias including aborted cardiac arrest in 31% of patients with isolated inferolateral MAD.^3^ In our cohort, 3 out of 29 patients (10%) with isolated inferolateral MAD and without MVP showed inferolateral replacement fibrosis. Thus, our findings support the perspective that inferolateral MAD may represent a distinct disease entity.

### Limitations

Limitations of our study include the cross-sectional study design, which describes an association between an accelerated motion of the basal inferolateral LV wall and local replacement fibrosis, whereas the association of replacement fibrosis with outcomes, such as ventricular arrhythmias and mortality remains elusive. The retrospective observational nature of our study does not allow to derive causality or to evaluate the directionality of the observed associations. We included participants referred for CMR imaging in Switzerland, therefore the results may not apply to the general population. A potential selection bias cannot be excluded, as patients were included based on report-based detection of MAD rather than systematic screening of the entire CMR population. Reports of CMR imaging studies were screened retrospectively for the presence of MAD and median MAD size was slightly larger than reported in previous studies^3,14^ suggesting that patients with more severe pathologies were involved in the current study. The length of inferolateral MAD was measured in 3-chamber view but the circumferential extension of the MAD, which may also affect movement pattern of the basal LV segments, was not assessed in the current study. Despite excellent inter-rater reliability, the movement pattern of the basal inferolateral LV segment was assessed by visual justification, which is prone to inter-observer variability.

## Conclusion

In conclusion we found that age and accelerated longitudinal motion of the basal inferolateral LV segment are independently associated with replacement fibrosis of the basal inferolateral LV wall, a well-recognized source of ventricular arrhythmias in patients with inferolateral MAD. Our observations for the first time provide a potential link between abnormal movement pattern of the basal inferolateral LV segment and the development of fibrosis. Furthermore, the presence of bileaflet MVP and MAD size were significantly associated with this specific LV movement pattern, suggesting that both, a pathological mitral valve apparatus and MAD are relevant determinants for motion abnormalities of the basal inferolateral LV segment.

## Supplementary Material

qyaf120_Supplementary_Data

## Data Availability

The data that support the findings of this study are available from the corresponding author upon reasonable request.

## Lead author biography

Martin F. Reiner, MD, PhD, is a cardiologist, specialist in internal medicine and currently fellow in the Rhythmology Department at the University Heart Center, University Hospital Zurich (Switzerland). He completed an MD–PhD program at the University of Zurich in basic science. His research focuses on thrombogenesis in cardio– and cerebrovascular disease and arrhythmogenic mitral valve prolapse, with an emphasis on translational approaches bridging basic science and clinical application.
